# Response-Locked Brain Dynamics of Word Production

**DOI:** 10.1371/journal.pone.0058197

**Published:** 2013-03-12

**Authors:** Stéphanie Riès, Niels Janssen, Borís Burle, F.-Xavier Alario

**Affiliations:** Aix-Marseille Université, CNRS, Marseille, France; Northwestern University, United States of America

## Abstract

The cortical regions involved in the different stages of speech production are relatively well-established, but their spatio-temporal dynamics remain poorly understood. In particular, the available studies have characterized neural events with respect to the onset of the stimulus triggering a verbal response. The core aspect of language *production*, however, is not perception but *action*. In this context, the most relevant question may not be how long after a stimulus brain events happen, but rather how long before the production act do they occur. We investigated speech production-related brain activity time-locked to vocal onset, in addition to the common stimulus-locked approach. We report the detailed temporal interplay between medial and left frontal activities occurring shortly before vocal onset. We interpret those as reflections of, respectively, word selection and word production processes. This medial-lateral organization is in line with that described in non-linguistic action control, suggesting that similar processes are at play in word production and non-linguistic action production. This novel view of the brain dynamics underlying word production provides a useful background for future investigations of the spatio-temporal brain dynamics that lead to the production of verbal responses.

## Introduction

Producing language is one of our most commonly used faculties. Everyday, we use speech for a variety of motivations and purposes. The common feature across all these situations is neither the motivation or purpose, but rather the speech act as well as the earlier cognitive processes that are required and lead to the encoding and production of spoken utterances. Neuropsychological and haemodynamic studies have provided a relatively detailed map of the brain regions involved in single word production, the most standard laboratory test case for investigating language production ([1]–[3], and below). Additionally, electrophysiological studies are providing an increasing body of evidence regarding the timing of the distinct processes required to produce single words ([4], and below).

However, in previous neurophysiological investigations of language production, neural events associated to linguistic processes have generally been characterized solely with respect to stimulus onset [5]. This stimulus-locked approach, presumably inspired by previous research on language processing where comprehension processes were at stake, is undoubtedly reinforced by the fear of electro-myographic (EMG) articulation artifacts. Although this approach has brought valuable information regarding the timing of various processes, it may be suboptimal to clearly dissociate the so-called “lead-in” processes linked to the identification of the stimulus (as defined by [5]), from actual linguistic processes. Indeed, the core aspect of language *production* is not perception but *action*. Words must be actively selected, articulated and monitored, irrespective of the internal or external stimulus that triggered speech production (e.g., a picture or an internal goal to communicate). In the context of laboratory experiments, the most relevant question may therefore not be how long after the stimulus brain events happen, but rather how long before the production act do they occur.

Our proposal here is to investigate language production-related brain activity not only time-locked to the stimulus but also, and most importantly, time-locked to vocal onset. Comparing brain activities from these two points of view has been successfully applied to disambiguate EEG activities linked to stimulus perception, response execution, and selection processes in EEG studies of action selection and control outside of language (e.g. [6], [7]) and in single-cell recordings in Macaque monkeys [8], [9]. There, activities linked to response execution emerge time-locked to the response, but are reduced, and possibly absent, time-locked to the stimulus presentation. The opposite is true for activities evoked by the stimulus. In addition, decision/selection-related activities are thought to occur at the crossroad between stimulus perception and response execution; they are expected to be visible both time-locked to stimulus presentation and to the response, although they will be less phasic than stimulus-evoked activities (i.e., these activities will not be as well averaged to the stimulus, thus their slopes will not be as steep and their amplitude will be less important as stimulus-locked components but they will also be less-transient, e.g., [8]).

Before reporting the word production experiment we conducted on the above premise(s), we briefly review the broader context for our study, namely the evidence available on the brain regions and temporal dynamics involved of word production. Producing a word requires a chain of cognitive processes. In the context of picture naming, often used in experimental settings, the picture must be recognized and the appropriate *concept* must be activated. Then, *linguistic processes* are needed to retrieve, select and encode the corresponding word, and finally *motor processes* come into play to articulate this word. Conceptual processes are subserved by a distributed network of areas including parieto-occipital and inferior temporal cortices (e.g., [10], [11]). Linguistic processes related to the retrieval and encoding of words are associated with regions of the left temporal cortex (e.g., [2], [12], [13], [14]), and also regions of the medial frontal and left inferior frontal cortex (e.g. [3], [15]–[19]). Finally, articulation processes have been associated with premotor and primary motor areas, as well as the somatosensory cortex and the posterior superior temporal lobe (e.g., [20]–[22]). Electrophysiological studies of word production suggest that lexical access starts around 200 ms after stimulus onset [23]–[26], [ but see also 27] and is seen best on the P2 component (i.e., positivity peaking around 200 ms post-stimulus onset, [25]). There is also some evidence that morphology affects word production around 350 ms post stimulus [28] (see also [29]). Finally, phonological encoding could start between 275 and 350 ms post-stimulus [30]–[33].

To our knowledge, research on the brain activities time-locked to the speech response is relatively infrequent. The available studies have focused on the investigation of motor preparation processes by considering voice-related cortical potentials (i.e., Bereitschaftpotential for speech) peaking around speech onset (e.g. [34]–[38], described more in the discussion), or on speech monitoring processes occurring after speech onset (e.g. the error-related negativity) [39]–[43]. Importantly, in these studies, many of the core linguistic processes that lead to the preparation and execution of a verbal response (see above) were not considered. To this end, describing activities time-locked to both the stimulus presentation *and* the vocal onset would seem to be critical. Studies involving such approach are exceedingly rare (with MEG: [44], [45]). Thus, the rationale of comparing surface neurophysiological activity leading to verbal responses with that evoked by the stimulus has yet to be explored. Of note, this approach has been recently used in the study of intracranial EEG recordings (e.g., [46]–[48]). Edwards and collaborators used electrocorticography (ECoG) to investigate word production [47]. They reported activities recorded directly from the cortical surface of the left inferior and medial frontal gyri (IFG & MFG), and left supra-marginal gyrus (SMG) peaking 600 ms after stimulus presentation and 200 ms before vocal onset. These locations are consistent with findings from imaging studies [5]. However, the spatial coverage of intracranial studies is limited as it is dictated by clinical needs (see [49] for a review of intracranial studies of language production, and its relative merits and limitations). Therefore, other brain areas may generate activities before vocal onset that are critical for word production, but may not have been detected with this technique.

In the current study, we used EEG to elaborate a comprehensive description of the spatio-temporal dynamics of brain activities leading to word production. We attempted to circumvent the relatively poor spatial resolution of monopolar EEG signal by estimating the current source density (Laplacian computation), which is known to enhance spatial resolution and to provide a good estimation of the corticogram [50]. In addition, we also estimated the cortical generators of the activities of interest by performing a distributed source localization, time-locked to both stimulus and vocal onset. We are thus able to obtain a finer spatial resolution than provided by monopolar EEG (as usually reported) in addition to the excellent temporal resolution inherent to this technique.

One main difficulty that arises in a response-locked analysis of speech production activities is that articulatory muscular activity, occurring at the time of interest, produces massive artifacts on the brain signal (e.g., [51]). Such EMG artifacts occur before and during response utterance, thus leaving a relatively short (and largely undetermined) time window of “EMG-free” signal between the presentation of the stimulus and the response. Different strategies have been elaborated to bypass EMG artifacts, including the downright avoidance of overt speech production (e.g. [32]). However, none of these strategies has provided the ability to observe a clean, undistorted signal on the whole time-window needed to overtly name a picture. We have recently put forward a tentative solution to address this issue, namely a blind source separation algorithm based on canonical correlation analysis [52]. This approach was efficient for revealing monitoring processes occurring after the vocal response: precisely when participants are articulating [43]. In the present study, we used the same methods to investigate what happens earlier, going backwards from the overt verbal response towards the stimulus that triggered it.

On the basis of the studies reviewed above, the time course of EEG activities elicited by picture naming should be as follows. The first components we expect to observe, time-locked to stimulus presentation, are the well-described visual evoked potentials (e.g. [53]) associated with perceptual processes. These will be presumably followed by components associated to conceptual access, which can be expected over the occipito-parietal junction and inferior temporal regions. Then we should observe components associated to linguistic processes over left temporal, medial frontal and left frontal regions. Importantly, these left temporal, medial frontal and left frontal components should be observed time-locked to both the stimulus and vocal onset. Valuable predictions in respect to medial frontal activities can be based on the comparison with the non-linguistic literature of action-control that inspired the current research. Indeed, a fronto-medial EEG component associated to *non-linguistic* response selection peaks 40 ms before the onset of response execution [54] (see also [6], [7], [55]) and is presumed to originate in the SMA and pre-SMA region [56]–[58]. Observing the same activity in word production (as suggested by fMRI studies of word selection [15], [18], [19]) would suggest a similar domain-general process is needed to produce words. Finally, components reflecting motor processes should be observed only time-locked to vocal onset. These should peak around vocal onset, when the response starts to be produced.

In short, we present a detailed and novel description of the EEG activities preceding vocal verbal responses by considering them not only locked to the stimulus, but also to the onset of verbal response. This is made possible by the strategy we adopted to overcome articulation-related EMG artifacts. Moreover, besides a mere description of the time course of activities, we aim at establishing a link between electrical activities and the structures generating them thanks to signal processing approaches enhancing the spatial resolution of EEG.

## Methods

### 1. Ethics statement

This study was approved by the local institutional review board (IRB: Comité d'éthique de l'Université de Provence, Aix-Marseille I). According to the declaration of Helsinki, written informed consent before the start of the experiment was obtained from each subject.

### 2. Participants

A total of 16 right-handed native French-speakers (7 females) with normal or corrected to normal vision participated in the experiment (mean age: 23.6). The data of 4 participants were removed from the behavioral and electrophysiological analysis due to problems during the EEG recordings (over 45% of the trials were rejected due to artifacts). Hence, the data of 12 participants (5 women) were analyzed.

### 3. Materials and Design

Forty-five line drawings of common objects (mean name agreement was 95.6%, σ = 6.96%, σ = Standard deviation) were used as stimuli [59] (for more information about the picture name properties, see Supplementary Materials S1, Tables S1 and S2 and Figure S2). They were all 11×11 cm and were presented centrally with a visual angle of 2.22°. Each of the 45 experimental items appeared in a pseudo-random order twice per block such that two consecutive items were semantically and phonologically unrelated. Overall, participants named each of the 45 different pictures 20 times. This unusually high number of repetition was chosen to reduce the variability between trials and to yield reliable estimates of the core electrophysiological components underlying the basic picture naming task (we show in the supplementary materials that the shape and topography of the components we describe are not affected by repetition: Figures S3 and S4). Participants performed an initial familiarization phase to get rid of any priming effect in the experiment-proper (see Procedure below).

### 4. Procedure

Participants were tested in a sound-attenuated dimly-lit environment. They were seated in a Faraday room in front of a computer screen. The experiment was controlled by the software Eprime 2.0 Professional (Psychology SoftwareTools, Inc., Pittsburgh, PA), which allows on-line recording and voice-key triggering of the participants' verbal responses. We used a piezzo-electric microphone.

A trial consisted of the following events: (1) a fixation point (“plus” sign presented at the center of the screen) for 500 ms; (2) a picture, which remained on the screen until the participants responded or until a 1500 ms deadline was reached (3) a blank screen for 1000 ms. Importantly, the picture disappeared when the subject's voice triggered the voice-key, stressing a reaction-time situation. The subsequent trial started automatically. An experimental run comprised 90 trials in which participants saw the whole set of pictures twice. There were 10 runs in the experiment. The participant's task was to name out loud, as fast and as accurately as possible, the picture presented. They were also asked to remain as relaxed as possible and to avoid making movements which could generate artifacts on the EEG recordings (e.g. eye blinks, frowning) during the experiment. Response latencies were measured from the onset of the stimulus to the beginning of the vocal response by means of a software voice key which sensitivity was adjusted to the voice of each participant (included in Eprime 2.0 Professional). Offline, the accuracy of this measure was checked visually and corrected when necessary using the software CheckVocal [60] which displays both the waveforms and the spectrograms of the utterances.

The experiment consisted of three parts. First, following a standard procedure in this task, participants were familiarized with the 45 pictures used in the experiment. The pictures were presented one by one in a random order, and the participant was asked to name each one of them. The experimenter made verbal corrections when an incorrect or unexpected response was produced. Second, the microphone sensitivity was tested and adjusted to the voice of the participant while (s)he was reading words presented on the screen. Third, the experimental instructions were delivered and the experiment started. The experimental session lasted for about an hour. There were short breaks between runs which length varied depending on how long the subject needed to rest.

### 5. Electrophysiological recordings

The EEG was recorded from 64 Ag/AgCl pre-amplified electrodes (BIOSEMI, Amsterdam) (10–20 system positions). The sampling rate was 512 Hz (filters: DC to 104 Hz, 3 db/octave). The vertical electrooculogram (EOG) was recorded by means of two surface electrodes just above and below the left eye, respectively. The horizontal EOG was recorded with two electrodes positioned over the two outer canthi.

### 6. Data pre-processing

#### 6.1. Behavioral data pre-processing

Trials were coded as errors when the participant produced any kind of verbal error: partial or complete production of incorrect words, verbal dysfluencies (stuttering, utterance repairs, etc.). Erroneous trials and trials where recording failures occurred, for example when the voice key triggered for a reason not linked to the participant's voice or when the participant did not answer within the 1500 ms limit, were removed from further analysis.

#### 6.2 EEG data pre-processing

After acquisition, the EEG data were filtered (high pass = 0.16 Hz). Eye movement artifacts were then corrected using the statistical method of Gratton, Coles and Donchin [61].

Speaking induces large facial EMG activities that contaminate the EEG signal. To reduce the EMG artifacts induced by articulation, we used a Blind Source Separation algorithm based on Canonical Correlation Analysis (BSS-CCA) [62] that separates sources based on their degree of autocorrelation. The suitability of BSS-CCA for removing articulatory EMG bursts from EEG signal is described in detail in [52] using the same data as in the present study. Components observed without BSS-CCA can be seen in Figures S5 and S6. We note that some of the described components described (notably at FT8, FC1 and FCz) were affected by the absence of BSS-CCA, underlying the importance of the use of this algorithm to observe the entirety of the components leading to word production. In the current application the BSS-CCA method was applied on non-overlapping consecutive windows of 1.5 seconds (corresponding to the maximum length of a trial) enabling the targeting of local EMG bursts, in contrast to tonic EMG activity produced by continuous contraction of the facial or neck muscles (this was done automatically using the EEGLAB plug-in Automatic Artifact Removal implemented by Gómez-Herrero available at http://www.cs.tut.fi/~gomezher/projects/eeg/software.htm#aar). EMG related components were selected according to their Power Spectral Density (PSD). As explained in [52], components were considered to be EMG activity if their average power in the EMG frequency band (approximated by 15–30 Hz) was at least 1/5 of the average power in the EEG frequency band (approximated by 0–15 Hz).

Following the BSS-CCA procedure, all other artifacts (left over EMG activity or blinks that were not well corrected) were rejected on the basis of a trial-by-trial visual inspection of monopolar recordings. We note that the use of the Laplacian transformation is very sensitive to small **local** artifacts (i.e., artifacts present at single electrodes: phasic artifacts as well as slow electrical shifts), they were thus also carefully rejected. The retained monopolar recordings were averaged, separately, to stimulus presentation and to vocal-onset. Laplacian transformation (i.e., current source density, C.S.D., estimation), as implemented in BrainAnalyser™ (Brain Products, Munich), was applied to each participant's average. One of the main advantages of the use of Laplacian transformation is that it is reference-free, contrarily to the more generally used monopolar recordings. In addition, Laplacian transformation is known to substantially improve the spatial definition of the monopolarly-recorded EEG signal [63]–[64], providing a good estimation of the corticogram [50], [65]. Importantly, this sharpening effect reveals temporal differences otherwise obscured by volume conduction [66]. Hence, as a side effect, improving the spatial resolution also, secondarily, improves the temporal separability of activities, and therefore the actual temporal resolution of EEG. The monopolar signal was first interpolated with the spherical spline interpolation procedure, fitting the head to the closest sphere. Then second derivatives in two dimensions of space were computed (Legendre polynomial: 15 degrees maximum; degree of spline: 3, [67]; We assumed a radius of 10 cm for the sphere representing the head, contrary to the unrealistic 1 m value implemented in BrainAnalyserTM. The resulting unit was µV/cm^2^. This change only affects the scale and not the topographies or any other aspect of the results). For averages time-locked to stimulus presentation baseline was taken as the 200 ms preceding stimulus presentation. For averages time-locked to vocal onset, the baseline was taken from 500 ms until 300 ms before vocal onset.

### 7. Analysis

Electrodes P9 and P10 were removed for all participants as they were too noisy for 1 of the 12 participants we kept in the analysis.

**Figure 1:**
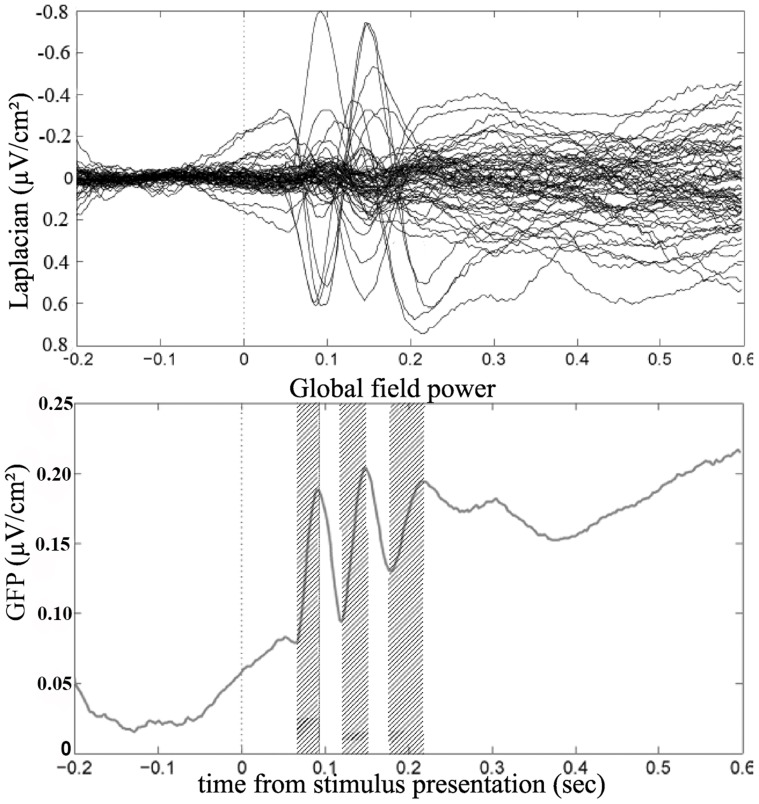
Butterfly plot (top) and global field power (bottom) of the Laplacian transformed data time-locked to stimulus presentation. The 3 hatched areas correspond to the three time-windows of interest, determined as corresponding to the three fastest increases of variance of the global field power: from 70 ms to 90 ms, from 125 ms to 150 ms, and from 185 ms to 220 ms post-stimulus presentation.

We describe the Laplacian-transformed EEG components time-locked to stimulus presentation and to vocal onset over the regions previously associated with picture naming by haemodynamic and neuropsychological studies: we therefore focused on activities recorded at occipital (electrodes Oz, O1, O2) and parieto-occipital (electrodes POz, PO3, PO4, PO8, PO7), posterior parietal (electrodes P7, P8, P5 and P6), left posterior temporal (electrode TP7 and contra-lateral electrode TP8), medial frontal (electrodes Cz, FCz, FC1 and FC2) and left inferior frontal electrodes (electrode FC5 and FT7 and contra-lateral electrodes FC6 and FT8). Although activities associated with linguistic processes involved in picture naming have often been described as left-lateralized, we also described activities observed at contra-lateral recording sites. We tested the statistical reliability of these activities across subjects by comparing to zero the slopes (assessed by a linear regression fit) of the Laplacian waveforms computed in the time-windows defined by the global field power (GFP, i.e. spatial standard deviation) [68] (Figure 1) (slope measures are independent of the chosen baseline and provide morphological information about the data [55]). The three time-windows corresponding to the fastest increase of variance on the GFP across electrodes were from 70 to 90 ms, from 125 to 150 ms and from 185 to 220 ms post-stimulus (Figure 1).

**Figure 2:**
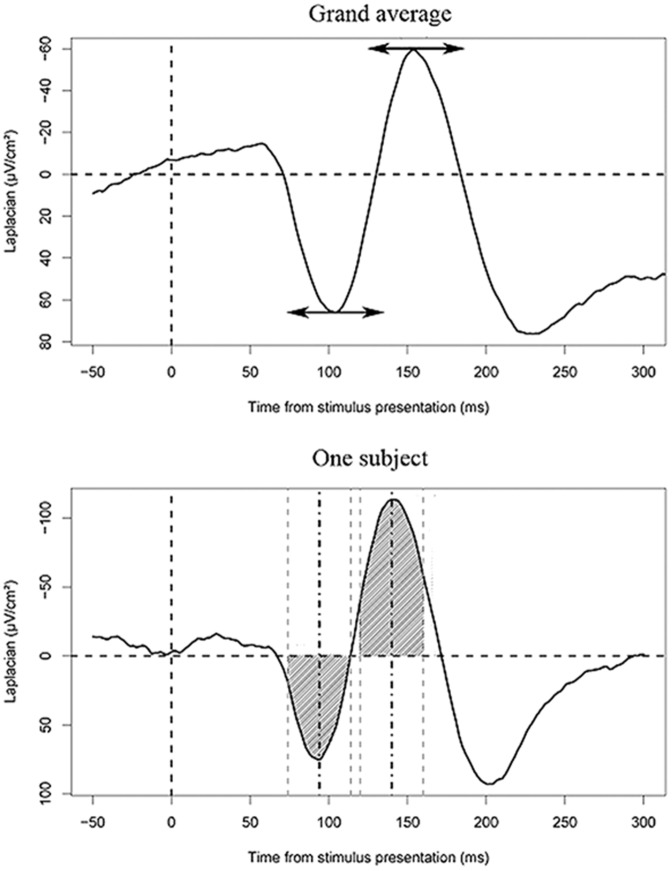
Measuring latencies and peak to peak amplitudes. Top: On the grand average, a fixed 100 ms time-window is defined around the latencies of the peaks of interest. Bottom: The latencies of the peaks of interest are measured for each participant on the time-windows previously defined on the grand averages. The peaks are defined as the maximum or minimum (for positivities and negativities respectively) of activity in these time-windows. This is done on smoothed data (using a moving average on 40 ms-long time-windows) to be as independent from the noise as possible. Importantly, the peaks were clearly identifiable on a subject-by-subject basis. Then, on the non-smoothed data, the surfaces (hatched areas) between the curve and the zero-line are measured on 40 ms-long time-windows (grey dotted lines) centered on the latencies of the peaks of interest (black dotted lines). Finally, to measure the amplitude of the rise of a peak, negative in this example, the difference between the surface measured around the negative peak and around the preceding positivity is computed. It is this surface difference that we refer to as the peak-to-peak amplitude.

The selected activities were then analyzed in more detail in the 700 ms following stimulus presentation and the 500 ms preceding vocal onset using two common measures known to be independent of the baseline: the latency of the peaks of interest and the peak-to-peak amplitude (i.e. the difference between the amplitude of two consecutive peaks of activity, see Figure 2 for more details).

All measures were compared using either Student's t tests or ANOVAs for comparisons of more than two groups. When ANOVAs were performed, the error term was always the interaction between the random factor Participants and the factor under analysis.

Although Laplacian computation dramatically improves the spatial resolution of EEG, it does not provide the generators of the identified activities. Moreover, Laplacian-transformed data are poorly sensitive to deep sources [69], [70]. To identify the generators of EEG activities, one needs to solve the so-called “inverse-problem” that relates scalp potential to cerebral sources. By itself, this problem has an infinite number of solutions, and thus additional constraints must be set to make it tractable. There are two classically distinguished families of inverse solutions: the so-called “distributed source” and the “equivalent dipole”approaches [71]. These two approaches rely on rather different hypotheses: distributed approaches are better suited for extended activities evolving in space and time. Because language related cortical activity has been described as prominently reflected in sustained activities that are not phase-locked to external events [49], we decided to focus on a distributed-type of model. To provide a more detailed view of the brain activity involved in picture naming, we also performed an equivalent dipole model that we describe in the Supplementary Materials S1 and Figure S1. We note this second type of model could only be performed time-locked to the stimulus.

We used a depth weighted minimum norm estimate [72], [73] using the software BESA Research 5.3 (MEGIS Software, Munich, Germany), computed on the envelope of the brain. The number of modeled dipoles was set to 800. Solving the inverse problem in this case reduces to a linear problem that amounts to estimate the amplitude of each dipole at every time point. The solution is thus a series of cortical maps of the each dipole intensity.

Two source models were constructed on the grand averages of the monopolar EEG signal time-locked, respectively, to stimulus presentation and to vocal-onset. Time-locked to stimulus presentation, the model was constructed on the first 650 ms after stimulus onset (i.e., corresponding to the average RT). Time-locked to vocal onset, the model was constructed on the 500 ms preceding vocal-onset. The baselines were the same as those used for the Laplacian analysis. Two types of weighting were applied: depth weighting and spatio-temporal weighting. Depth weighting was applied so that deep sources would not appear smeared in the minimum norm reconstruction but more focal as sources located closer to the cortical surface do. Spatio-temporal weighting was applied so that larger weight would be applied to sources more likely to contribute to the recorded data. This contribution is estimated following the signal subspace correlation measure introduced by [74]. The channel noise correlation matrix was estimated in the time-window defined as baseline.

Although tools are now available for testing differences, in amplitude or in power, between inverse solution of different experimental conditions, simply testing the statistical significance of the reconstructed activity in a given region is a much less explored question. In the present report, we estimated the null hypothesis (H0) statistics using the distribution of amplitudes of all the dipoles at each time point in the baseline (corresponding to the 200 ms preceding stimulus onset). This provides a distribution of amplitudes when no meaningful activity is present. Based on this H0 distribution, post-stimuli activities were considered as significant if their amplitude was higher than the *n*th quantile of the H0 distribution. To take into account multiple comparisons, we chose .001 as a statistical threshold.

## Results

We will present the behavioral data, followed by the Laplacian components observed chronologically. We will first present which components were clearly visible stimulus-locked and then those which were visible both time-locked to the stimulus and to vocal onset. Importantly, we will highlight what this new way of looking at language production EEG activities brings to their understanding. Finally, we will present the results of the surface minimum norm models time-locked to both events.

### 1. Behavioral results

The average Reaction Time (RT) for correct trials was 651 ms (σ = 72 ms). The average error rate was 1.31% (σ = 0.96%) (errors defined in “Materials” section). 0.48% of the trials were removed from further analysis due to no responses or voice key problems. Only the correct trials were included in further analysis (for an analysis of errors, see [43]).

### 2. Surface EEG components

After a trial by trial inspection of the EEG data, 72% (σ = 7%) of the trials were left for further analysis time-locked to stimulus presentation and 75% (σ = 7%) time-locked to vocal onset.

#### 2.1. Stimulus evoked potentials

**Figure 3:**
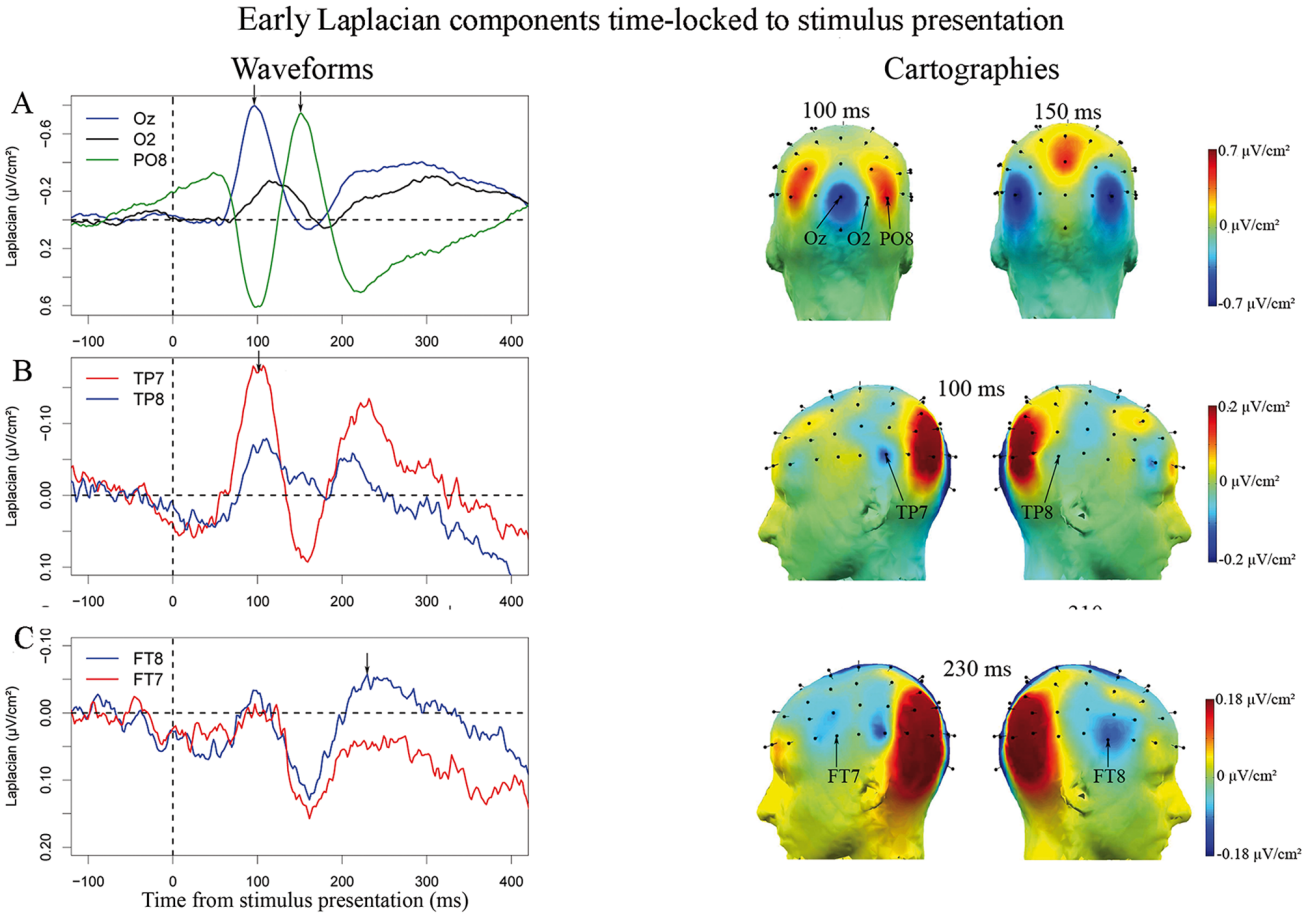
Early Laplacian Components time-locked to the stimulus: Surface Laplacian waveforms and cartographies. The scales vary across rows depending on the amplitude of the activity of interest. The vertical arrows on the waveforms indicate the latencies at which the cartographies on the right are presented. The chosen baseline is the 200 ms before stimulus presentation. **A.** Visual-evoked potentials at Oz, O2 and PO8. A negativity peaking 100 ms post-stimulus is observed for Oz. At the same latency, a positivity is observed for PO8 which may correspond to the same dipole of activities seen from the opposite side. The evolution of spatial distribution of these activities as illustrated by the topographies, supports this description. Later, a negativity peaks between 120 and 130 ms post-stimulus onset at O2. The polarity of the signal then reverses for PO8 and a negativity is observed peaking at about 150 ms post-stimulus. The pattern described is symmetrical: very similar waveforms were observed at the left homologue recording sites, O1 and PO7, than at O2 and PO8 (see Table 1 for statistical tests made on the slopes of these activities). **B.** The activity at TP7 peaks on average 98 ms post-stimulus presentation (σ = 19 ms), and is much larger than at TP8. **C.** The negativity recorded at FT8 peaked on average 239 ms post-stimulus onset (σ = 29 ms). No activity was significantly present at the contra-lateral site, FT7. We note that posterior activities are larger at this later latency. This may be explained by the increased depth of the underlying sources and/or by the existence of multiple neighboring generators. Indeed, Laplacian transformation is less sensitive to deep sources and those will appear smeared on the cortical surface. In addition, Laplacian transformation cannot separate foci of activity on nearby recording sites.

Time-locked to stimulus presentation, the Laplacian-transformed EEG data revealed a sequence of activities. A set of occipital and parieto-occipital activities identified as visual-evoked potentials were observed. We analyzed the activities observed at all electrodes situated over the occipital cortex and its junction with the parietal and temporal cortices: Oz, O1 and O2 (over the occipital cortex), POz, PO3, PO4, PO7 and PO8 (at the junction between the occipital cortex and the parietal cortex), and P5, P6, P7 and P8 (over the posterior parietal cortex). We observed a temporal sequence in the latencies at which the first negativity reached its maximum across these recording sites. The averaged latency of the first peak of activity was greater with increasing distance from the central electrodes (Oz & POz) (F(1,11) = 507.4; p<.001, Figure 3A and Table 1). No lateralization effect was found on the latency of this first negative peak (F(1,11) = 1.43, p = .26; central electrodes Oz and POz removed from the analysis). The topographies of these activities also reflect this posterior-anterior sequence. Indeed, at 100 ms post-stimulus onset, which corresponds to the average latency at which the activity recorded at Oz reaches its maximum, the focus of negativity is centered on Oz. Positivities at PO7 and PO8 are visible at this same latency suggesting the same symmetrical dipoles are being seen from opposite ends. The symmetrical shape of the waveforms recorded at Oz and PO7/PO8 in this early time-window further supports this interpretation. At 150 ms post-stimulus, an opposite pattern of activity is observed: negativities at PO7 and PO8 and a positivity at Oz. This suggests different dipoles of activity are now visible.

**Table 1:** Latency of the first peak of activity at posterior recording sites.

	left			center	right		
Relative distance	3	2	1	0	1	2	3
Dorsal electrodes:	P5:	PO3:		POz:		PO4:	P6:
Mean latencies (ms):	159 (11)	146 (26)		97 (10)		142 (13)	144 (17)
Ventral electrodes:	P7:	PO7:	O1:	Oz:	O2:	PO8:	P8:
Mean latencies (ms):	161 (8)	155 (12)	130 (29)	99 (9)	123 (20)	153 (9)	174 (15)

Latency of the 1^st^ peak of activity in ms for the electrodes Oz, POz, O1, O2, PO7, PO8, PO3, PO4, P7, P8, P5 and P6. The latency increases with increasing distance from the central recording sites. Oz and POz are at the mid-line, their “relative distance” is coded (0). P7, P5, P6 and P8 are the furthest, their “relative distance” is coded (3).

A clear early peak of activity was observed over the left temporal cortex, at electrode TP7 (Figure 3B), peaking in average 98 ms (σ = 19 ms) post-stimulus onset. At the contra-lateral site, TP8, a much smaller peak of activity was observed. The slope of the left activity at TP7 on the first time-window of interest was significantly different from zero (t(11) = −5.08; p<0.001) which was not the case for the right activity, at TP8, (t(11) = −1.87; p = 0.09). As a side note, the TP7 activity peaks in average 98 ms (σ = 19 ms) post-stimulus onset, in the same time-window as the earliest visual-evoked potential (e.g., at Oz). This early latency suggests that this activity is not included within the progression of activities described above but occurs independently. The peak of activity at TP7 is reached before the peak of activity at P7 (which occurs 161 ms, σ = 8 ms, post-stimulus presentation), while P7 is located upstream from TP7 on the visual pathway. Thus, activity over TP7 is not the negative pole of a dipole located between TP7 and PO7. Consistent with this view, the cartographies on Figure 3B show a small negativity centered on TP7. As mentioned earlier, it is the neighboring posterior large positivity (around PO7 and PO3) that very likely corresponds to the positive pole of a dipole recorded between the central posterior site (Oz) and PO7 and PO3.

An activity was observed in the next time-window over the right frontal cortex, at FT8 (Figure 3C). It peaked on average 239 ms post-stimulus onset (σ = 29 ms), with a slope significantly different from zero on the time-window spanning from 185 ms to 220 ms post-stimulus (t(11) = −4.28; p<0.01). At the contra-lateral site, at FT7, the amplitude of the second negativity was much smaller and its slope was not significantly different from zero (t(11) = −1.2, p = 0.26).

We note the components above-described (i.e., sequence of visual evoked potentials, early left posterior temporal and right frontal components) were no longer observable time-locked to the response.

#### 2.2. Fronto-medial and left frontal activities - Stimulus-locked

Fronto-medial activities were observed starting around 200 ms and peaking about 310 ms after stimulus presentation (slopes were significantly different from zero between 185 and 220 ms post stimulus at Cz, t(11) = −6.18, p<0.001; FCz, t(11) = −5.01, p<0.001; FC1, t(11) = −3.07, p<0.05; and FC2, t(11) = −2.49, p<0.05).

**Figure 4:**
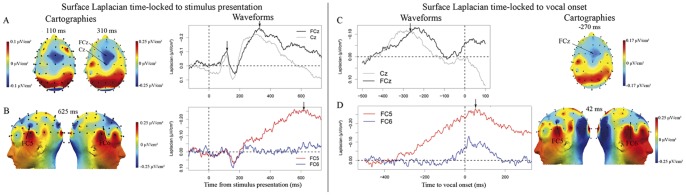
Later frontal components time-locked to stimulus presentation (A and B) and to vocal-onset (C and D): Surface Laplacian waveforms and cartographies. **A.** At fronto-central sites, there was a first negativity peaking on average 104 ms (σ = 19 ms) post-stimulus onset and a second larger one peaking around 300 ms post-stimulus onset. **B.** The activity at FC5 continued rising until about 600 ms after stimulus-onset, whereas the slope stayed flat at the contra-lateral recording site, FC6. **C.** Fronto-central activities at FCz and Cz peaking between 300 and 200 ms before vocal onset. **D.** Left frontal negativity at FC5 starting to rise about 350 ms before vocal onset and peaking on average 42 ms (σ = 116 ms) post vocal onset. A much smaller activity is visible at the contra-lateral site (FC6), it starts to rise much later than at FC5 (at about 100 ms before vocal onset) and peaks around the same latency as the negativity at FC5. The slope of the activity at FC6 (on the 100 ms preceding vocal onset) is only marginally different from zero (t(11) = −1.35, p = .10).

The evolution of the topographies from 200 ms to 300 ms indicates the emergence of a negativity from the central site, Cz, towards more frontal sites and especially FCz (Figure 4A, see cartography at 310 ms). The latency at which the negativity reached its maximum was significantly different between these two recording sites (t(11) = 2.18, p = 0.05, average at Cz: 281 ms, σ = 58 ms; average at FCz: 328 ms, σ = 32). We note also a small first negativity peaking around 100 ms after stimulus presentation at these fronto-medial recording sites (Figure 4A). The slope of these first activities were significantly different from zero on the time-windows spanning from 70 to 90 ms post-stimulus (Cz, t(11) = −5.13, p<0.001; FCz, t(11) = −3.45, p<0.01; FC1, t(11) = −3.42, p<0.01; FC2, t(11) = −3.45, p<0.01) and from 125 to 150 ms post-stimulus, except for the activity recorded at FC2 (Cz, t(11) = 4.93, p<0.001; FCz, t(11) = 3.08, p<0.05; FC1, t(11) = 2.69, p<0.05; FC2, t(11) = 1.37, p = 0.2).

A left frontal slow-rising negative activity was also observed (at FC5; Figure 4A). The slope of this activity was significantly different from zero on the last time-window of interest, from 185 to 220 ms post-stimulus (t(11) = −3.97, p<0.01). There was no clear peak of activity at this recording site time-locked to stimulus presentation but rather a continuous negative slope that reached its maximum roughly around 600 ms after stimulus presentation. The local topography shown around this time point reveals a negative activity centered around FC5 but not around the contra-lateral site, FC6. Indeed at FC6, no late negativity could be observed (slope analysis: t(11) = −1.13, p = 0.28).

#### 2.3. Fronto-medial and left frontal activities – Response-locked

We analyzed the EEG components at the same recording sites on the 500 ms preceding the vocal response. Fronto-central activities peaked around 250 ms before vocal-onset. Their slopes were significantly different from zero on the time-window corresponding to the 100 ms preceding the average latency of the negative peak (Cz: t(11) = −2.09, p<.05; FCz: t(11) = −1.87, p<.05; FC1: t(11) = −2.37, p<0.05; FC2: t(11) = −2.09, p<0.05, one-tailed student t tests were used as a negativity was expected given previous reports by [6], [7], [54], [55] Figure 4C). Contrary to the fronto-medial activities observed time-locked to stimulus presentation, there was now only one clear negative peak. The local topographies above the medial-frontal region look equivalent time-locked to both events, suggesting similar underlying activities were observed (see Figure 4A vs. 4C). Similarly to what we reported time-locked to the stimulus, the peak at Cz preceded the peak at FCz. This temporal dynamic effect was however not significant across subjects (t(11) = 1.58, p = 0.143).

We also observed a left frontal activity (at FC5) starting to rise about 350 ms before vocal onset and peaking on average 42 ms (σ = 116 ms) post vocal onset. The slope of the activity was significantly different from zero on a time-window spanning from 300 ms before vocal onset to vocal onset (t(11) = −2.23, p<.05). This was not the case at FC6 (t(11) = −0.55, p = .30, one-tailed student t tests were used as a negativity was expected at FC5 given the previous report by [75], Figure 4D). For this component, the peak was much clearer time-locked to the response compared to stimulus presentation. Here too, the local topographies were very similar time-locked to both events.

#### 2.4. Relationship between fronto-medial peak latencies and mean response times

Following a reviewer's suggestion, we estimated the relationship between response times and fronto-medial peak latencies (at electrodes FCz, Cz, FC1 and FC2) at the level of mean participant performance. We performed two types of tests: a) independent correlation tests between average peak latencies for each electrode and RT, and b) a linear regression model with RT as dependent variable and the peaks of activities at the aforementioned electrodes as predictors.

When the peaks were estimated stimulus-locked, there was no significant relationship between their latency and response times (correlation tests: FCz: ρ = 0.075, t(10)<1; Cz: ρ = 0.462, t(10) = 1.65, p = 0.131; FC1: ρ = 0.442, t(10) = 1.56, p = 0.150; FC2: ρ = 0.478, t(10) = 1.72, p = 0.116; linear regression model F(5–6) = 1.19, p = .41, all predictor t's<1, for Cz: t(10) = 1.78, p = .12 ).

When the peaks were estimated response locked, there was a significant relationship between their latency and response times. The independent correlation analysis suggested the relationship may be true at various electrodes (FCz, ρ = −0.908, t(10) = −6.87, p<0.001; FC2, ρ = −0.664, t(10) = −2.81, p<0.05), marginally so at Cz (ρ = −0.552, t(10) = −2.10, p = 0.063) and non-significant at FC1 (ρ = −0.172, t(10) = −0.55, p = 0.59). The grouped linear regression confirmed the robustness of the relationship at FCz (models F(5–6) = 5.965, p = .035, adjusted R2 = .73; effect of FCz t = −2.64, p = .046, all other t's<1).

Summarizing, the analysis conducted at the level of average participant performance reveals an absence of significant correlation for stimulus-locked peaks and a significant correlation for response-locked peaks.

### 3. Exploring cortical sources

**Figure 5:**
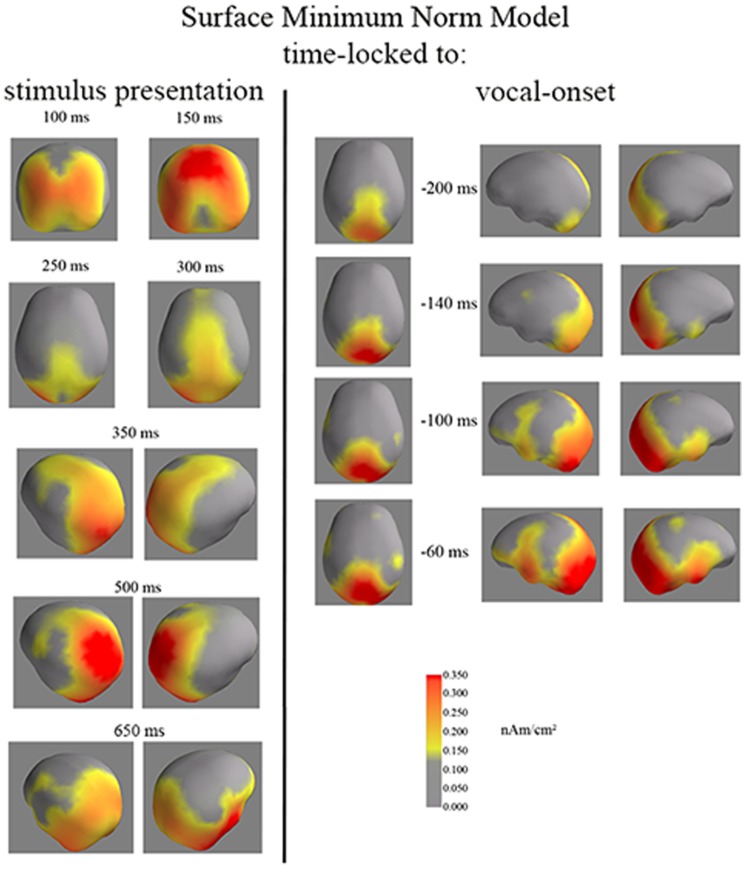
Surface Minimum Norm time-locked to stimulus presentation (left) and to vocal onset (right). The surface minimum norm images display the occipital and fronto-central activities as well as the left-frontal activity from 100 ms until 650 ms after stimulus presentation and from 200 ms until 60 ms before vocal onset. Time-locked to vocal-onset, the fronto-central activity decreases while the left frontal activity increases. The fronto-central activity is visible at −270 ms (as on the cartographies of the Laplacians) but is less clear than at −200 ms. The left frontal activity starts being visible −140 ms and stays left lateralized until −75 ms. A subject by subject analysis of the significance of activity in this region and contra-lateral one confirms the left frontal activity is present before activity at the contra-lateral site.

Time-locked to stimulus presentation, the surface minimum norm model revealed early bilateral occipital activity (100 ms post-stimulus), followed by more extended occipito-parietal activity (150 ms post-stimulus), corresponding to the extent of the activities on the cartographies of the Laplacian data (Figure 5). The fronto-medial activity was clearly visible from about 250 ms post-stimulus onwards (in contrast, the activity observed at 110 ms after stimulus presentation in the Laplacian was not significantly present on the minimum norm reconstructions; it was only visible as a very small increase on the equivalent dipole model described in the supplementary materials). The evolution of the activities from 200 ms to 300 ms indicates the emergence of a negativity at central sites followed by a shift towards more frontal and lateral sites. The fronto-central activity then starts decreasing from 350 ms post-stimulus onwards. At 350 ms post-stimulus, a left frontal activity starts and its intensity increases until the end of the time-window. No activity was visible on the right frontal cortex. The early left occipito-temporal activity observed on the Laplacian data (electrode TP7) is not accounted for as a distinct activity in the minimum norm solution. However, the posterior bilateral activity seems to be more pronounced on the left side than on the right starting around 150 ms post-stimulus onset and continuing until the end of the time-window of interest (see Figure 5 at 350, 500 and 650 ms after stimulus onset and Movie S1). Since the visual evoked EEG potentials themselves were not lateralized, the more pronounced left posterior activity may reflect the left temporal activity visible at TP7 on the Laplacians. Indeed, given the small amplitude of the left temporal EEG component compared to that of the visual evoked potentials, these could have acted as an attraction basin in the source localization solution. The dipolar model described in the supplementary materials is in agreement with this hypothesis.

Time-locked to vocal onset, the surface minimum norm model reveals both a fronto-central activity and a left frontal activity (Figure 5). Similarly to what we have described time-locked to stimulus presentation, the fronto-medial and left frontal activities develop sequentially: The fronto-central activity emerges first. It is present from −285 ms to −60 ms before the vocal-onset but starts decreasing as the left frontal activity starts being visible around −150 ms before vocal onset. The left frontal activity increases all the way to vocal onset. No activity is visible on the contra-lateral site until −75 ms before vocal-onset. Moreover, at this late latency, the lateral activity is much less focal on both hemispheres (see Movie S2 for further details).

## Discussion

The aim of this study was to provide novel insight on the brain processes underlying language production by looking at EEG activities not only time-locked to stimulus presentation, as usually performed, but also, preceding and time-locked to vocal onset. We were able to overcome the problem posed by articulation-related EMG artifacts, and thus report clear activities time-locked to stimulus onset *and* to vocal onset. Laplacian transformation and source modeling of the EEG signal constrained the possible origin of the time-resolved activities. In addition to posterior and frontal components time-locked to stimulus presentation, we report medial-frontal and left frontal components preceding vocal-onset. These activities have different time-courses and likely stem from cortical regions pointed out in previous brain imaging studies of picture naming.

### 1. Fronto-medial and left frontal activities tied to vocal-onset

Unprecedentedly in the study of language production, we report two prominent EEG components *both* time-locked to stimulus presentation *and* to the vocal response. These activities differed on their spatial and timing properties. The fronto-central activities were recorded on electrodes FCz, FC1, FC2, and Cz, and localized in the medial frontal gyrus by source modeling (see also dipole #5 in dipolar model in the supplementary materials). They peaked around 300 ms after stimulus presentation, and around 270 ms before vocal onset. They then decreased to reach baseline just before vocal onset. The left frontal activity was recorded on electrode FC5, and localized around the left middle/inferior frontal areas by source modeling. It started rising around 350 ms post-stimulus, reaching its maximum shortly after vocal-onset. Distributed source models performed both time-locked to stimulus presentation and to vocal onset show that the left frontal activity starts when the medial frontal activity is at its maximum and increases until vocal onset as the medial frontal activity decreases. To our knowledge, this constitutes the first report of such sub-second dynamic interplay between frontal EEG components preceding vocal onset in speech production. The functional role of these novel activities can be hypothesized by comparing them to earlier observations in EEG and imaging studies.

The peak of the fronto-medial EEG activities appeared to be equidistant from the stimulus onset and the vocal onset. Moreover, the order in which the peaks of the activities at Cz and FCz occurred on the grand averages was the same time-locked to both events, and the local topographies were very similar. However, correlation analyses performed between the latencies of the medial frontal peaks and mean reaction times suggested a larger temporal variability in the processes occurring between the stimuli and frontal peaks than those occurring between frontal peaks and the responses. These observations suggest that these activities reflect a process at the cross-road between stimulus perception and response execution and closely linked to the emission of the response. In the early MEG study by [44], a comparable activity was reported. It was accentuated when observed time-locked to vocal-onset compared to stimulus presentation. A very similar set of EEG activities has been previously associated with response selection in non-linguistic tasks and similarly associated with pre-SMA activity [6], [7], [54], [55]. This activity is present in tasks involving at least two alternative responses but not in simple RT tasks, where there is only one possible response to be made [54]. The very similar activity observed here suggests an extension of the interpretation made by these authors to a case where there is a much greater variety of possible responses. Moreover, our results suggest this medial frontal activity may reflect a process common to language and other cognitive functions. This conclusion is in line with the observations made in imaging studies (e.g., [15], [18], [19]). The temporal relationship between response-locked peaks and response times provides a further argument for linking these frontal activities to response-related processes (i.e., response selection or preparation), although it is difficult to be more specific given the available evidence. We note that activity in medial frontal regions is not included in the model proposed by [5] contrarily to that proposed by [3].

The left-frontal activity was clearly better observed time-locked to the response compared to stimulus presentation as it reached its maximum just around vocal onset. This suggests this activity can be functionally linked to the execution/production of the response rather than to the perception of the stimulus. In agreement with this observation, MEG and intracranial studies of picture naming have reported late activities, starting no earlier than 300 ms after stimulus presentation, in the left frontal cortex (MEG: [44], [76]–[78]; ECoG: [45]). Occasionally, these frontal activities were described time-locked to vocal-onset [44], [47]. Edwards and collaborators [47] also described different foci of left frontal activities with different time-courses in picture naming. The more posterior frontal activities peaked around vocal onset, similarly as in our study. In respect to functional interpretation, two possibilities come to mind. One potential interpretation for this activity and that at the contra-lateral recording site comes from the literature on the Bereitschaftspotential (or “readiness potential”) associated to motor preparation for speech execution [34]–[38]. This potential is described as a slow rising frontal negativity peaking at the onset of the execution of volitional oral movements. Although the Bereitschaftspotential was initially described as left-lateralized in speech [79], later investigations of this potential seem to converge in indicating it is in fact bilateral [34], [36]–[38]. In our study, lateral frontal activity time-locked to vocal onset was clearly larger and started earlier over the left hemisphere. However, a right frontal activity is also visible at the contra-lateral recording site starting around 100 ms before vocal onset. Thus the later part of the left frontal potential and the right potential may reflect motor preparation processes. A plausible interpretation of the early onset of the left frontal activity could be tied to phonological encoding. Previous evidence suggest phonological encoding starts around 350 ms after stimulus presentation [30]–[33]. This corresponds roughly to the onset of the left frontal activity we report. Moreover, the posterior left frontal region has been linked to phonological encoding and/or syllabification by fMRI (e.g., [21], [80]; see [3] for a review) and MEG studies of picture naming [77] (see also [5], [81] for meta-analyses).

We are aware that both the medial and left frontal regions have been associated to specific processing stages of word production. For example, anterior portions of the left IFG (pars orbitalis) have been associated to higher-order semantic processing [82], the posterior left IFG has been linked to the resolution of competition between lexical representations [16], [17], [83]–[86], and the SMA has been linked to speech monitoring [87]–[88]. Our study does not enable us to tease apart the relative contributions of sub-regions within the medial and the left frontal cortices. This is, in part, because of the limited spatial resolution of EEG compared to fMRI or MEG. Most importantly, this is because we intentionally aimed to provide a general overview of the activity underlying word production, without specifically targeting one or another of these processes by means of specific experimental contrasts. In this context, what our results show, for the first time, is that medial frontal activity *precedes* left frontal activity in picture naming, thus constraining the functional interpretation of these activities. The medial frontal activity is likely tied to response selection or response preparation processes whereas the left frontal activity seems more closely linked to phonological encoding and subsequent response execution processes initiated around 350 ms before vocal onset, when the word to be produced has been chosen. These conclusions find support in the mesial-lateral organization suggested in word production [15], and previously in non-linguistic cognitive control [89], whereby the medial frontal cortex triggers activity in the lateral frontal areas to enable regulatory adjustments for action.

### 2. Stimulus-locked components

While our focus is on response-locked components, some aspects of the stimulus-locked activity deserve discussion. In the paradigm we used, word production starts with the identification of the depicted object, and linking it with a known concept. The Laplacian data revealed the signature of these initial processes in the form of successive early components at posterior sites (i.e., Oz, POz, PO8, PO7, etc; see e.g., [53]). There was a clear spatio-temporal sequence in the visual evoked potentials, whereby activity spread symmetrically and bilaterally from central to lateral posterior sites. These results extend those described in relation to the identification of simple geometric-shaped stimuli [90] to more complex and variable line-drawings. Source modeling of visual evoked potentials provides specific indications about their possible origin: initially in the secondary visual cortices, and then in the occipito-parietal junction (see equivalent dipole model in the supplementary material). These two areas have been associated, respectively, with visual and conceptual processing (e.g., [1]).

The data also reveal an early left posterior temporal activity, peaking 100 ms after stimulus presentation. Source modeling however did not capture this activity independently. This could be explained by the vicinity of the visual-evoked potentials which were of much higher amplitude than the lateralized temporal activity and could have acted as an attraction basin in the surface minimum norm and the dipolar source solution. In agreement with this suggestion, the amplitude of the left posterior modeled sources are larger than their right homologues, while the actual visual-evoked potentials visible on the Laplacian data are not lateralized. The left middle and posterior temporal cortex is linked to lexical access in multiple haemodynamic and neuropsychological picture naming studies [2], [91]–[93] (see also [26]). We note however that other EEG studies focused on the timing of lexical access and selection generally report later latencies (around 200 ms after stimulus presentation, [23]–[25]). This difference may be due to methodological discrepancies between our data analysis methods and theirs, and further investigations specifically targeting lexical access should be performed to clarify this issue.

### 3. Conclusions

We described a detailed sequence of activities that occur prior to overt speech production in a simple picture naming task. The temporal interplay of the fronto-medial and left frontal activities is consistent with the organization described in non-linguistic action control, suggesting that similar processes are at play in word production and non-linguistic action production. We interpret the earlier fronto-medial activity as a reflection of word selection processes, and the later left frontal activity as tied to word production processes. Together, these findings constitute a novel view of the brain dynamics underlying word production. The methodology and specific hypotheses put forward in this study provide a useful background for future investigations of the spatio-temporal brain dynamics that lead to the production of verbal responses.

## Supporting Information

Table S1 **Effects of pictures and picture names' properties on the peak to peak amplitudes around the first negative peak observed at the listed electrodes and on the latency of this negativity time-locked to stimulus onset.** The first peak to peak amplitude corresponds to the rise of the negativity whereas the second peak to peak amplitude corresponds to its resolution. The apparent word length effect at early posterior sites is presumably due to a confound with image complexity discovered post-hoc. The data for which the difference between conditions was significant are highlighted in yellow and those for which the difference was marginally significant are highlighted in light yellow.
(DOC)

Table S2 **Effects of picture names' and pictures properties on the peak to peak amplitude (corresponding to the rise of the negativity) of the second negative peak observed at the listed electrodes and on its latency time-locked to stimulus presentation.** The data for which the difference between conditions was significant are highlighted in yellow and those for which the difference was marginally significant are highlighted in light yellow.
(DOC)

Figure S1 **Equivalent dipole model.** The dipoles' waveforms, locations, Talairach coordinates, and C.S.D. maps of the model are displayed. This model explained 93% of the variance. The C.S.D. maps of the model are presented at the latencies at which C.S.D. maps of the data were presented in Figure 3. The scale of the figures can vary depending on the activity depicted.
(EPS)

Figure S2 **Effect of Picture Name Lexical Frequency and Picture Name Length on TP7, PO4 and FC2 time-locked to stimulus presentation and FCz time-locked to vocal onset.** The level of significance of the effects is indicated by asterisks: *: p<0.05. **A.** At TP7, low frequency picture names induced a greater amplitude than high frequency ones at the rise of the negativity peaking on average 98 ms (σ = 19 ms) post-stimulus. The latency of the maximum of negativity was not significantly affected by lexical frequency (t(11) = 1.75, p = .11) The length of the picture names had no effect on these amplitude and latency measures (ts<1). **B.** High frequency picture names induced a greater amplitude at the positivity at PO4, peaking on average 217 ms (σ = 26 ms) post-stimulus. **C.** Bisyllabic words induced a greater amplitude of the negativity observed at FC2 than monosyllable ones. Latencies were not affected by Picture Name Length. There was no effect of Picture Name Frequency on the amplitude of the fronto-central negativity, but the negativity peaked later for low frequency picture names than for high frequency ones at Fz (not shown because not visible on the grand averages, see text for details). **D.** Bisyllabic words induced a greater amplitude at the resolution of the negativity than monosyllabic words on FCz.
(EPS)

Figure S3 **Surface Laplacian waveforms and cartographies in 10 first repetitions versus 10 last repetitions for visual-evoked potentials at Oz, O2 and PO8 (top), left-posterior temporal component at TP7 and contra-lateral waveform at TP8 (middle), and right inferior frontal component at FT8 and contra-lateral waveform at FT7 (bottom).**
(EPS)

Figure S4 **Surface Laplacian waveforms and cartographies in 10 first repetitions versus 10 last repetitions for fronto-medial components at FCz (top) and Cz (middle), and for fronto-lateral components at FC5 and FC6 (bottom).** Although repetition seemed to have an effect on the fronto-medial and the lateral frontal components, these effects were not significant.
(EPS)

Figure S5 **Surface Laplacian waveforms and cartographies before BSS-CCA for visual-evoked potentials at Oz, O2 and PO8 (A), left-posterior temporal component at TP7 and contra-lateral waveform at TP8 (B), and right inferior frontal component at FT8 and contra-lateral waveform at FT7 (C).**
(EPS)

Figure S6 **Surface Laplacian waveforms and cartographies before BSS-CCA for fronto-medial components at FCz and Cz (A and C), and for fronto-lateral components at FC5 and FC6 (B and D).**
(EPS)

Movie S1 **Surface minimum norm time-locked to stimulus presentation from −200 ms until 500 ms after.** The 200 ms preceding the stimulus presentation correspond to the chosen baseline. The activities are shown in nAm/cm^2^.
(AVI)

Movie S2 **Surface minimum norm time-locked to vocal onset from −500 ms until vocal onset.** The first 200 ms correspond to the chosen baseline. The activities are shown in nAm/cm^2^.
(AVI)

Supplementary Materials S1 Response-locked brain dynamics of word production.
(DOC)
