# Supplementary material for: Response-Locked Brain Dynamics of Word Production
Source: PLoS One. 2013 Mar 12;8(3):e58197. doi: 10.1371/journal.pone.0058197 (PMC3595260; doi:10.1371/journal.pone.0058197)
Supplement: Table S1 — Effects of pictures and picture names' properties on the peak to peak amplitudes around the first negative peak observed at the listed electrodes and on the latency of this negativity time-locked to stimulus onset. The first peak to peak amplitude corresponds to the rise of the negativity whereas the second peak to peak amplitude corresponds to its resolution. The apparent word length effect at early posterior sites is presumably due to a confound with image complexity discovered post-hoc. The data for which the difference between conditions was significant are highlighted in yellow and those for which the difference was marginally significant are highlighted in light yellow. (DOC) [file pone.0058197.s001.doc]

Table S1

Effects of picture names' and pictures properties on the first activities of interest.

|  | **1st peak to peak amplitude (µV/cm²)** | | | | **2nd peak to peak amplitude (µV/cm²)** | | | | **Latency 1st negativity (ms)** | | | |
| --- | --- | --- | --- | --- | --- | --- | --- | --- | --- | --- | --- | --- |
|  | **Frequency:** | | | | | | | | | | | |
| **Electrode:** | high | low | t-test | p | high | low | t-test | p | high | low | t-test | p |
| Oz | 0.32 | 0.37 | -1.99 | 0.072 | 0.38 | 0.41 | -1.56 | 0.146 | 98 | 99 | -0.89 | 0.392 |
| O1 | 0.16 | 0.17 | -0.64 | 0.537 | 0.13 | 0.17 | -1.60 | 0.137 | 145 | 140 | 0.97 | 0.352 |
| O2 | 0.22 | 0.29 | -2.12 | 0.057 | 0.23 | 0.26 | -0.84 | 0.421 | 118 | 124 | -1.30 | 0.221 |
| POz | 0.14 | 0.15 | -0.48 | 0.640 | 0.31 | 0.33 | -1.12 | 0.286 | 97 | 97 | 0.00 | 1.000 |
| PO3 | 0.37 | 0.39 | -0.41 | 0.688 | 0.57 | 0.51 | 1.79 | 0.100 | 142 | 148 | -1.14 | 0.279 |
| PO4 | 0.43 | 0.44 | -0.51 | 0.618 | 0.59 | 0.54 | 2.33 | 0.040 | 142 | 141 | 0.42 | 0.684 |
| PO7 | 0.59 | 0.6 | -0.59 | 0.564 | 0.72 | 0.68 | 1.27 | 0.231 | 154 | 156 | -1.26 | 0.233 |
| PO8 | 0.58 | 0.64 | -1.52 | 0.157 | 0.65 | 0.64 | 0.30 | 0.773 | 154 | 155 | -0.23 | 0.822 |
| P5 | 0.2 | 0.2 | 0.46 | 0.654 | 0.21 | 0.2 | 1.65 | 0.126 | 157 | 160 | -1.62 | 0.133 |
| P6 | 0.15 | 0.16 | -0.27 | 0.790 | 0.24 | 0.21 | 1.72 | 0.113 | 153 | 161 | -3.22 | 0.008 |
| P7 | 0.26 | 0.27 | -1.22 | 0.248 | 0.23 | 0.25 | -1.42 | 0.184 | 162 | 164 | -0.53 | 0.607 |
| P8 | 0.19 | 0.18 | 0.26 | 0.801 | 0.25 | 0.24 | 0.56 | 0.588 | 179 | 171 | 0.92 | 0.376 |
| TP7 | 0.1 | 0.13 | -2.44 | 0.033 | 0.13 | 0.13 | 0.13 | 0.896 | 111 | 104 | 1.75 | 0.107 |
|  | **Length:** | | | | | | | | | | | |
|  | bisyll | msyll | t-test | p | bisyll | msyll | t-test | p | bisyll | msyll | t-test | p |
| Oz | 0.36 | 0.31 | 3.00 | 0.012 | 0.41 | 0.36 | 1.77 | 0.104 | 98 | 98 | 0.28 | 0.785 |
| O1 | 0.21 | 0.15 | 2.39 | 0.036 | 0.15 | 0.13 | 1.22 | 0.246 | 135 | 123 | 1.56 | 0.147 |
| O2 | 0.23 | 0.22 | 0.31 | 0.763 | 0.24 | 0.23 | 0.83 | 0.422 | 122 | 118 | 0.71 | 0.495 |
| POz | 0.14 | 0.15 | -0.39 | 0.701 | 0.31 | 0.31 | -0.13 | 0.898 | 95 | 98 | -0.88 | 0.396 |
| PO3 | 0.41 | 0.33 | 2.61 | 0.024 | 0.55 | 0.55 | -0.15 | 0.882 | 147 | 146 | 0.45 | 0.661 |
| PO4 | 0.47 | 0.38 | 3.13 | 0.010 | 0.59 | 0.55 | 1.69 | 0.119 | 139 | 139 | 0.40 | 0.699 |
| PO7 | 0.63 | 0.54 | 3.82 | 0.003 | 0.73 | 0.69 | 1.62 | 0.134 | 156 | 154 | 1.11 | 0.289 |
| PO8 | 0.63 | 0.55 | 2.75 | 0.019 | 0.68 | 0.61 | 2.43 | 0.033 | 156 | 151 | 2.91 | 0.014 |
| P5 | 0.2 | 0.19 | 0.54 | 0.598 | 0.22 | 0.21 | 0.36 | 0.725 | 162 | 162 | -0.07 | 0.948 |
| P6 | 0.17 | 0.15 | 0.96 | 0.356 | 0.25 | 0.23 | 0.57 | 0.580 | 152 | 151 | 0.04 | 0.973 |
| P7 | 0.27 | 0.28 | -0.55 | 0.595 | 0.23 | 0.23 | 0.45 | 0.660 | 162 | 163 | -0.12 | 0.910 |
| P8 | 0.19 | 0.2 | -0.27 | 0.789 | 0.26 | 0.24 | 0.74 | 0.473 | 180 | 177 | 0.74 | 0.478 |
| TP7 | 0.12 | 0.1 | 0.69 | 0.502 | 0.14 | 0.14 | 0.26 | 0.801 | 100 | 101 | -0.40 | 0.700 |
|  |  |  |  |  |  |  |  |  |  |  |  |  |
| Post-hoc analysis: **Image Complexity** | | | | | | | | | | | | |
| **Electrode:** | high | low | t-test | p | high | low | t-test | p | high | low | t-test | p |
| Oz | 0.4 | 0.3 | 2.71 | 0.020 | 0.43 | 0.37 | 1.83 | 0.095 | 99 | 97 | 0.84 | 0.418 |
| O1 | 0.23 | 0.13 | 2.29 | 0.043 | 0.18 | 0.11 | 2.14 | 0.055 | 131 | 126 | 1.62 | 0.133 |
| O2 | 0.3 | 0.22 | 2.34 | 0.039 | 0.27 | 0.22 | 1.68 | 0.122 | 127 | 114 | 1.90 | 0.085 |
| POz | 0.16 | 0.13 | 1.30 | 0.220 | 0.34 | 0.3 | 2.58 | 0.026 | 97 | 94 | 1.18 | 0.263 |
| PO3 | 0.41 | 0.36 | 1.24 | 0.242 | 0.54 | 0.53 | 0.67 | 0.516 | 143 | 147 | -0.83 | 0.426 |
| PO4 | 0.46 | 0.41 | 2.47 | 0.031 | 0.54 | 0.57 | -1.30 | 0.221 | 141 | 141 | -0.16 | 0.875 |
| PO7 | 0.62 | 0.57 | 2.23 | 0.048 | 0.73 | 0.67 | 1.82 | 0.096 | 157 | 153 | 2.04 | 0.066 |
| PO8 | 0.65 | 0.57 | 1.65 | 0.127 | 0.65 | 0.64 | 0.33 | 0.745 | 155 | 153 | 1.56 | 0.147 |
| P5 | 0.21 | 0.19 | 0.82 | 0.429 | 0.2 | 0.22 | -1.20 | 0.255 | 163 | 155 | 2.34 | 0.039 |
| P6 | 0.16 | 0.16 | -0.19 | 0.857 | 0.22 | 0.23 | -0.37 | 0.720 | 148 | 154 | -0.67 | 0.515 |
| P7 | 0.26 | 0.26 | 0.14 | 0.892 | 0.25 | 0.28 | -1.02 | 0.329 | 163 | 162 | 0.48 | 0.643 |
| P8 | 0.18 | 0.18 | -0.21 | 0.840 | 0.23 | 0.24 | -0.36 | 0.727 | 178 | 172 | 0.51 | 0.622 |
| TP7 | 0.13 | 0.11 | 0.79 | 0.448 | 0.14 | 0.14 | -0.39 | 0.703 | 98 | 100 | -0.63 | 0.541 |
