# Supplementary material for: Response-Locked Brain Dynamics of Word Production
Source: PLoS One. 2013 Mar 12;8(3):e58197. doi: 10.1371/journal.pone.0058197 (PMC3595260; doi:10.1371/journal.pone.0058197)
Supplement: Table S2 — Effects of picture names' and pictures properties on the peak to peak amplitude (corresponding to the rise of the negativity) of the second negative peak observed at the listed electrodes and on its latency time-locked to stimulus presentation. The data for which the difference between conditions was significant are highlighted in yellow and those for which the difference was marginally significant are highlighted in light yellow. (DOC) [file pone.0058197.s002.doc]

Table S2

Effects of picture names' and pictures properties on the second activities of interest.

|  | **peak to peak amplitude (µV/cm²)** | | | | **Latency of negative peak (ms)** | | | |
| --- | --- | --- | --- | --- | --- | --- | --- | --- |
|  | **Frequency:** | | | | | | | |
| **Electrode:** | high | low | t-test | p | high | low | t-test | p |
| FCz | 0.18 | 0.16 | 1.08 | 0.303 | 332 | 306 | 2.05 | 0.065 |
| FC1 | 0.12 | 0.11 | 0.53 | 0.604 | 292 | 324 | -1.74 | 0.111 |
| FC2 | 0.15 | 0.15 | -0.13 | 0.899 | 323 | 334 | -0.52 | 0.614 |
| Cz | 0.18 | 0.19 | -0.25 | 0.807 | 301 | 286 | 1.18 | 0.265 |
| FT8 | 0.11 | 0.12 | -0.77 | 0.459 | 248 | 244 | 0.29 | 0.774 |
| TP7 | 0.13 | 0.13 | 0.61 | 0.553 | 212 | 214 | -0.24 | 0.812 |
|  | **Length:** | | | | | | | |
|  | bisyll | msyll | t-test | p | bisyll | msyll | t-test | p |
| FCz | 0.17 | 0.19 | 0.74 | 0.475 | 333 | 319 | 1.40 | 0.190 |
| FC1 | 0.1 | 0.11 | 0.41 | 0.693 | 292 | 302 | -0.80 | 0.439 |
| FC2 | 0.12 | 0.18 | 2.67 | 0.022 | 321 | 305 | 1.32 | 0.214 |
| Cz | 0.19 | 0.19 | 0.32 | 0.752 | 303 | 275 | 1.77 | 0.105 |
| FT8 | 0.1 | 0.14 | 1.79 | 0.100 | 247 | 238 | 0.53 | 0.607 |
| TP7 | 0.12 | 0.15 | 1.64 | 0.130 | 223 | 221 | 0.15 | 0.884 |
|  |  |  |  |  |  |  |  |  |
| Post-hoc analysis: **Image Complexity** | | | | | | | | |
| **Electrode:** | high | low | t-test | p | high | low | t-test | p |
| FCz | 0.18 | 0.16 | 0.80 | 0.439 | 322 | 309 | 1.12 | 0.288 |
| FC1 | 0.11 | 0.12 | -0.59 | 0.564 | 310 | 305 | 0.30 | 0.767 |
| FC2 | 0.14 | 0.16 | -2.05 | 0.065 | 306 | 318 | -0.79 | 0.448 |
| Cz | 0.19 | 0.18 | 0.49 | 0.634 | 268 | 290 | -1.45 | 0.176 |
| FT8 | 0.13 | 0.1 | 1.28 | 0.225 | 239 | 235 | 0.47 | 0.648 |
| TP7 | 0.14 | 0.11 | 1.50 | 0.162 | 223 | 214 | 0.88 | 0.396 |
