# Supplementary material for: Response-Locked Brain Dynamics of Word Production
Source: PLoS One. 2013 Mar 12;8(3):e58197. doi: 10.1371/journal.pone.0058197 (PMC3595260; doi:10.1371/journal.pone.0058197)
Supplement: Supplementary Materials S1 — Response-locked brain dynamics of word production. (DOC) [file pone.0058197.s011.doc]

Supplementary materials section for:

“Response-locked brain dynamics of word production”

Stéphanie Riès^1,2^ (corresponding author), Niels Janssen^1,3^, Borís Burle^1^, F.-Xavier Alario^1^

1: Aix-Marseille Université, CNRS, Marseille, France.

Full address: Pôle 3C, Centre St Charles. 3, place Victor Hugo. 13331 Marseille Cedex 03. FRANCE

E-mail addresses:

[stephanie.ries@berkeley.edu](mailto:stephanie.ries@berkeley.edu)

[njanssen@ull.es](mailto:njanssen@ull.es)

[boris.burle@amu](mailto:boris.burle@univ-provence.fr).fr

[francois-xavier.alario@amu](mailto:francois-xavier.alario@univ-provence.fr).fr

2: Present address: Helen Wills Neuroscience Institute, University of California Berkeley, 210 Barker Hall, Berkeley, California, 94703-3190, USA. Lab phone: 510-643-9744, Lab fax: 510-642-3192.

3: Present address: Universidad de La Laguna, Facultad de Psicología, Campus de Guajara, La Laguna, S/C de Tenerife, 38205 – España.

I. Equivalent dipole modeling

**1.1. Methods**

In the “equivalent dipole” approach, one assumes that, at a given point in time, brain activity can be *summarized* by a limited number of dipoles whose position, orientation and amplitude need to be specified. Since the number of variables is rather limited (6 * *N*, 6 being the number of free parameters per dipole, i.e., 3 position parameters, 2 angles defining direction and 1 amplitude parameter, *N* being the number of hypothesized dipoles), the problem becomes tractable. One difficulty, however, is to define the correct number *N*. In the present study, we used the Laplacian-transformed data and the surface minimum-norm model to determine the likely number of dipoles and the time-windows in which to fit them. A source model for the evoked potentials was constructed using the software BESA Research 5.3 (MEGIS Software, Munich, Germany). This model was performed on the grand averages time-locked to stimulus presentation. Activities being less phasic time-locked to vocal-onset, this type of modeling appeared to be less adequate.

The model was constructed on the 500 ms post stimulus-onset. Based on the time-course of the surface EEG activities, we placed the dipoles successively on the three time-windows corresponding to the fastest increase of variance on the GFP of the monopolar data (as the model was based on the monopolar data). These correspond to the same time-windows as defined on the Laplacians except for the last window which is somewhat longer (70-90 ms, 125-150 ms and 185-300 ms, the 3rd time-window as defined by the GFP performed on the Laplacian (185-220 ms) was to narrow to fit the last dipole). When fitted, equivalent dipoles were allowed to vary on orientation, amplitude and position. Depending on the Laplacian components observed and surface minimum norm model, a symmetry constrain could be added on the Position parameter. To further establish the validity of the model, we also report the topography of the current source density of the model at the time-points when the Laplacian components reached their maximum. This provides a further check that the model not only fits the surface potential function but also its second spatial derivative.

**1.2. Results**

Based on the current source density estimates, we constructed a model comprising two pairs of symmetric dipoles over occipital and occipito-parietal areas and an extra fronto-central one (see Figure S1). We thus followed a stepwise modeling procedure. The first pair of dipoles, constrained to be symmetrical, was fitted in the first time-window defined from the GFP (70 to 90 ms post-stimulus). This resulted in a bilateral localization in secondary visual cortices, around middle occipital gyrus, BA 18). The waveforms peaked 100 ms after stimulus presentation, at the same latency as the Laplacian waveforms observed at Oz. At the peak of these activities, the residual variance was 3%. Once fitted, this first pair of dipole was fixed, and not fitted anymore on the subsequent time windows. The second pair of dipoles, also symmetrically constrained, was fitted in the second time-window (125 to 150 ms post-stimulus). Their waveforms peaked 150 ms after stimulus presentation, at about the same latency as the Laplacian waveforms observed at PO8, PO7, PO3 and PO4. Their localization converged towards the parieto-occipital sulcus / posterior cingulate region (BA 30). At the peak of these activities the residual variance was 1%. The amplitude of the left dipole of this pair was larger than its right homologue, as shown by a comparison across subjects of the surfaces below the waveforms of these dipoles during the time-window of interest (t(11) = 4.0, p < 0.01). Since the visual evoked potentials themselves were not lateralized, the larger amplitude for the left dipole may reflect the left temporal activity visible at TP7 on the Laplacians, the two activities being merged into a single dipole by the algorithm. The location of the last dipole, fitted in the 3^rd^ time-window (185 to 300 ms post-stimulus), converged in the medial frontal gyrus (central part of BA 6, Figure S1B). The waveform of this dipole showed a first small dip peaking 111 ms post-stimulus and reached its first plateau at about 300 ms after vocal-onset, which corresponds to the time-range at which the negativities observed at fronto-central sites reached their maximum on the Laplacians. At the peak of this last activity the residual variance was 5%. The total residual variance after fitting all 5 equivalent dipoles to the stimulus-locked grand average ERP data was 7%. The C.S.D. cartographies of the model (Figure S1) are very similar to the C.S.D. cartographies of the data (Figure 3A & 3D) at all three latencies, even though the model was fitted on the monopolar data and *not* on the Laplacians. We note a slight difference in shape for the C.S.D. cartography made at 300 ms post-stimulus onset, whereby the real data presents a more elongated shape. This is likely due to a property of the data not modeled by a single dipole. The medio-frontal activity seems to shift from central to more frontal electrodes (see above); modeling this shift would require the dipole to move, or the addition of a second dipole, with a slightly different time course. However, given the spatial proximity of these two dipoles, the model would likely merge them in a single one.

The lateralized activities described on the Laplacians at TP7, FT8 and FC5 were not independently accounted for by the model. However, these activities are much smaller and isolated than the neighboring visual evoked potentials. Their overall variance may be too small to be modeled by distinct dipoles.

II. Picture names' properties

**2.1. Methods**

The lexical frequency and length of the picture names were controlled. While the expected effects did not stem out on the behavioral data, some of the EEG components we have reported in the main article were modulated by these properties. We are thus reporting this aspect of the results in the supplementary materials only, as it may help us gain some insights of the functional meaning of the components.

There were 18 high lexical frequency and 27 low lexical frequency picture names. Eighteen of these objects' names were monosyllabic, 18 were bisyllabic and 9 were trisyllabic. Half of the monosyllabic and of the bisyllabic names were of high lexical frequency; all the trisyllabic names were of low lexical frequency as high frequency trisyllabic names are very rare. Lexical frequency was not correlated with the visual complexity of the pictures (as rated in [1]; Spearman's correlation: S = 1576.71; p = .80 ).

**2.2. Results**

2.2.1. Behavioral data

A mixed effect linear regression analysis [2] conducted on the inverse transformed naming latency reaction time data revealed a marginal effect of Word Length (t(10599)=1.77, p = .07), where RTs increased with increasing word length. Log transformed Lexical Frequency did not affect RTs (t(10599) < 1). These analysis were performed using the package “languageR” (available at <http://cran.r-project.org/web/packages/languageR/index.html>) for the statistical software R (available at: http://www.r-project.org).

2.2.2. Electrophysiological data: Time-locked to stimulus presentation

Peak-to-peak amplitude (see Figure 2), latency measures and the corresponding statistical tests are reported in Tables S1 and S2.

Low lexical frequency picture names induced a larger negativity at TP7 (t(11) = -2.44, p <.05, peaking on average 98 ms post-stimulus, σ = 19ms, Figure S2A) and a smaller positivity at PO4 (t(11) = 2.33, p <.05, peaking on average 217 ms post-stimulus, σ = 26 ms Figure S2B). The first negativity at P6 (peaking on average 144 ms post-stimulus, σ = 17 ms) reached its maximum later for low lexical frequency picture names than for high lexical frequency ones (t(11)=-3.22, p<.01, not shown). Importantly, the latency of the preceding positive dip was not affected by Lexical Frequency at P6 (t(11) =1.67, p =0.12).

The second negativity at FC2 started to rise at about 180 ms post stimulus onset and reached its maximum on average 311 ms (σ = 44 ms) post stimulus onset; it was larger for bisyllabic picture names (t(11) = 2.67, p < 0.05) than for monosyllabic ones. The negativity at FT8, peaking on average 239 ms (σ = 29 ms) post-stimulus onset, also tended to be larger for bisyllabic picture names than for monosyllabic ones (t(11) = 1.79, p = 0.10). At a number of occipital electrodes, bisyllabic words induced larger visual evoked potentials than monosyllabic ones. However the variable length appeared to be confounded with the complexity of the image as defined in [1] (1999; see Table S1 for details).

In summary, we observed early lexical frequency effects on parieto-occipital and posterior temporal sites. There were later word length effects on fronto-medial and a trend at right frontal sites.

2.2.3. Electrophysiological data: Time-locked to vocal-onset

There was no effect of Picture Name Frequency on the amplitude nor on the latency measures of the fronto-central negativities. However, the slope of the negativity at FC5 (on the 300 ms preceding vocal onset) was marginally steeper for low frequency picture names than for high frequency ones (t(11)=1.93; p = 0.08, we did not perform analysis on the amplitude of the activity as there was no clear preceding positivity to enable measuring the peak to peak amplitude. Instead, we performed analysis on the slope of the activity on the time-window used to assess its presence. ). Bisyllabic picture names induced a greater amplitude than monosyllablic ones at the resolution of the negativity peaking around 250 ms before vocal onset at FCz (t(11)= 2.81, p< .05, see Figure S2). There was no effect of Picture Name Length on the latency at which the fronto-central negativities reached their maximum nor on the slope of the negativity at FC5 (t(11)=-1.38, p=0.20).

1. Repetition effects

As each item was repeated 20 times, we checked whether the shape and the topography of the components we described were affected by this factor. We separated the data into the first 10 repetitions and the last 10 repetitions to check for an effect of repetition. As shown in Figure S3 and S4, the shape and locus of the components we describe in the manuscript do not change with repetition. Although the amplitude of the frontal activities seemed affected by repetition on the grand averages, this effect was not significant (analysis performed on response-locked components at FCz, t(11)=1.24, p=0.24; and at FC5, t(11)<1; we performed the statistics on the same measures on which we tested for an effect of lexical frequency and length of the picture names for each component: peak-to-peak amplitude for FCz and slope of the rising negativity at FC5). Most importantly, we show that the activities we report time-locked to the stimulus and the response are still present in the second half of the experiment and are not significantly affected by repetition.

1. Description of activities observed without BSS-CCA

We performed the same averages after the exact same pre-processing steps excluding the rejection of muscle-related artifact with BSS-CCA. The same activities could generally be observed although the signal-to-noise ratio was lower for lateral activities both time-locked to the stimulus and to the response (Figure S5 B and C and Figure S6 B and D). Notably, posterior temporal and lateral frontal activities at 100 and 230 ms after stimulus presentation appeared much less lateralized on the grand averages (waveforms and topographies). The statistical analyses revealed the slope of the described left-posterior temporal negativity peaking in average 98 ms post-stimulus was still statistically different from zero on the first time-window of interest (t(11)=-4.99; p<0.001) and not its right homologue (t=-1.79; p=0.10), as described after BSS-CCA. However, the slope of the right frontal activity peaking in average 239 ms post-stimulus was no longer significantly different from zero (t(11)<1) on the third time-window of interest (from 185 to 220 ms post-stimulus) when no BSS-CCA was performed (the slope of its left homologue was still not significantly different from zero, t(11)<1).

The left frontal component described at FC5 and peaking around 600 ms after stimulus presentation was not statistically affected by the absence of BSS-CCA. Its slope was still statistically different from zero on the 3^rd^ time-window of interest when no BSS-CCA was performed (t(11)=-3.47; p<0.01). The fronto-medial activities peaking around 100 ms and 300 ms after stimulus presentation were hardly affected by the absence of BSS-CCA, only the slope at FC1 was no longer significantly different from zero on the second time-window of interest (from 70 to 90 ms post-stimulus: FCz: t(11)=-3.51, p<0.01; FC1: t(11)=-3.78, p<0.01; FC2: t(11)=-2.84, p<0.05; Cz: t(11)=-4.39, p<0.01; from 125 to 150 ms post-stimulus: FCz: t(11)=3.18, p<0.01; FC1: t(11)=1.44, p=0.18; FC2: t(11)=0.46, p=0.65; Cz: t(11)=4.91, p<0.001; from 185 to 220 ms post-stimulus: FCz: t(11)=-4.55, p<0.001; FC1: t(11)=-2.52, p<0.05; FC2: t(11)=-2.78, p<0.05; Cz: t(11)=-6.23, p<0.001).

Time-locked to vocal onset, the absence of BSS-CCA had a statistical effect on the fronto-medial component observed at FCz, its slope was no longer different from zero on the 100 ms preceding the latency of the maximum of the peak (t(11)=-1.66; p=0.06; one-tailed Student t-tests were used as described in the main manuscript). The other activities described were still statistically significant despite the absence of BSS-CCA on the same time-windows as described in the main manuscript (FC1: t(11)=-5.38, p<0.001; FC2: t(11)=-2.24, p<0.05; Cz: t(11)=-3.64, p<0.01; FC5: t(11)=-1.81, p<0.05).

References:

1. Alario F-X, Ferrand L (1999) A set of 400 pictures standardized for french: Norms for name agreement, image agreement, familiarity, visual complexity, image variability, and age of acquisition. Behav Res Methods Instrum Comput 31: 531 – 552.
2. Baayen HR, Davidson DJ, Bates DM (2008) Mixed effects modeling with crossed random effects for subjects and items. J Mem Lang 59: 390-412.
